# Pro-angiogenic effect of IFNγ is dependent on the PI3K/mTOR/translational pathway in human retinal pigmented epithelial cells

**Published:** 2010-02-10

**Authors:** Baoying Liu, Lisa Faia, Mengjun Hu, Robert B. Nussenblatt

**Affiliations:** Laboratory of Immunology, National Eye Institute, National Institutes of Health, Bethesda, MD

## Abstract

**Purpose:**

To investigate the molecular signaling pathway of Interferon gamma (IFNγ) contributing to angiogenesis in retinal pigmented epithelial (RPE) cells and the role of Phosphoinositide 3-kinase (PI3K)/mammalian target of rapamycin (mTOR) in this process.

**Methods:**

Human adult and fetal RPE cells were used in this study. Real-time polymerase chain reaction was used to detect human vascular endothelial growth factor (VEGF) mRNA expression. Thiazolyl blue tetrazolium bromide (MTT) assay was used to detect cell viability. VEGF expression from cell supernatant was measured using enzyme-linked immunosorbent assay (ELISA). Small interfering RNA (SiRNA) of signal transducers and activators of transcription 1 (stat1) and protein kinases B (akt) were transfected into ARPE-19 cells to directly study the roles of these molecules in VEGF expression. Sodium dodecyl sulfate PAGE (SDS–PAGE) and western blot analysis were used to detect the expression of signaling molecules.

**Results:**

IFNγ promoted human VEGF expression in both adult and fetal RPE cells. The PI-3K/Akt/mTOR/p70 S6 kinase pathway is required for IFNγ-induced VEGF expression in retinal cells. The mTOR inhibitor, rapamycin, along with the SiRNA targeted to akt and the PI3K inhibitor, LY294002, decreased hVEGF secretion from RPE cells. Moreover, IFNγ-induced hVEGF expression was not affected by SiRNA targeted to Stat1, implying that the classic Jak-Stat1 pathway of IFNγ may not be involved in this process.

**Conclusions:**

We provide evidence that IFNγ induces VEGF expression in human retinal pigment epithelial cells. Our work emphasizes that the activation of the PI-3K/mTOR/translational pathway is important for IFNγ-mediated VEGF expression in RPE cells. By elucidating molecular signaling involved in this process, our findings provide further mechanistic insight into the successful clinical application of rapamycin therapy for choroidal neovascularization in age-related macular degeneration (AMD) and uveitis.

## Introduction

Angiogenesis is the result of a net balance between the activities exerted by positive and negative regulators [1]. Mounting evidence strongly suggests that the immune system plays an important role in angiogenesis. Pro-inflammatory cytokines, such as Interferon gamma (IFNγ); interleukin-6 (IL-6); tumor necrosis factor alpha (TNFα); Interleukin 1 beta (IL-1β) are the major cytokines in the pathogenesis of ocular inflammatory diseases. Many of the proinflammatory cytokines are involved in inflammatory angiogenesis [2–5]. However, IFNγ is thought to be an anti-angiogenic cytokine, due to its inhibitory effect on endothelial cell growth and capillary formation [1,6–8].

The classic signaling events induced by IFNγ are through the Janus kinase (Jak) pathway and the signal transducer and activator of transcription 1 (Stat1) pathway. The cascade of signal transduction is initiated upon the binding of dimeric IFNγ to its receptor, followed by the activation of the receptor-associated Jak1 and Jak2, which in turn phosphorylate Stat1. Stat1 then translocates into the nucleus and functions as a transcription factor. Beyond the Jak/Stat1 pathway, IFNγ can also activate the mitogen activated protein kinase (MAPK) and Phosphoinositide 3-kinase (PI3K) pathways [9]. The roles of these pathways in IFNγ-induced biologic effects, however, have not been clearly defined.

We recently reported treating a patient with multifocal choroiditis associated with choroidal neovasculization (CNV) with rapamycin, an mammalian target of rapamycin (mTOR) inhibitor [10]. CNV occurs in many intraocular inflammatory diseases such as uveitis, age-related macular degeneration (AMD), and others. Among them, AMD is the leading cause of blindness in the United States for people over 60 years old. In the wet, or exudative, AMD, choroidal blood vessels grow through Bruch’s membrane into the subretinal space, causing CNV, and resulting in accumulation of blood beneath the retina. CNV is present in only 10% of patients with AMD, but is responsible for 90% of cases with severe vision loss [11]. Vascular endothelial growth factor (VEGF) is known to play an important role in this process. It has been associated with choroidal neovascular membranes, the retinal pigment epithelium (RPE), and maculae with AMD [12]. Intraocular delivery of anti-VEGF therapies is now widely accepted as a treatment for the wet form of AMD. The pro-inflammatory cytokine IFNγ plays an important role in the pathogenesis of intraocular inflammatory disease. In the aqueous and vitreous of uveitis patients, IFNγ can reach levels greater than 100 pg/ml [13,14]. Mounting evidence supports the notion that AMD may be an inflammation-driven disease [1,6–8]. Although there are no studies showing the increased expression of IFNγ in AMD patients, it has been reported that IFNγ induces complement factor H (CFH) expression from RPE cells, implying that IFNγ has a potential role in AMD pathogenesis [15]. In this study, we provide evidence that IFNγ promoted human VEGF secretion from human RPE cells. Unexpectedly, IFNγ-induced hVEGF secretion was not through the classic IFNγ Jak/Stat1 pathway, but through the PI-3K/mTOR/p70-S6K-translational regulation pathway.

## Methods

### Cell culture

ARPE-19 cells (ATCC, Manassas, VA) were routinely cultured in Dulbecco’s Modified Eagle’s Medium (DMEM) supplemented with 10% fetal bovine serum (FBS), penicillin, streptomycin (100 U/ml each), and 2 mM L-glutamine (Invitrogen, Carlsbad, CA). Human fetal RPE (hfRPE) cells were kindly provided by Section on Epithelial and Retinal Physiology of National Eye Institute. hfRPE cells were cultured in Minimum Essential Medium Alpha (MEM-α) modified medium with additional supplements, as described previously [16]. Only passage 1 was used. Both primary hfRPE and an adult RPE cell line, ARPE-19, were used to detect IFNγ-promoted VEGF mRNA expression by real time PCR. In the subsequent experimental settings, only ARPE-19 was used. The cells were cultured in the presence or absence of 25 ng/ml IFNγ, 10 ng/ml TNFα, 50 ng/ml IL-6, and 12.5 ng/ml transforming growth factor β1 (TGFβ1).

### Real-time polymerase chain reaction

Total RNA was extracted from confluent monolayers of hfRPE (P1) and ARPE-19 cells (RNeasy Kit; Qiagen, Valencia, CA). Total RNA (100 ng-1 µg) was reverse-transcribed to cDNA (ReactionReady^TM^ First Strand cDNA Synthesis Kit, SABiosciences, Frederick, MD). RNA samples were tested for VEGF gene expression by using the quantitative real time PCR (RT–PCR) MasterMix from SABiosciences, following their RT–PCR manual. Real-time PCR was performed in triplicate, using a 96-well format PCR array and an ABI 7500 real-time PCR unit (Applied Biosystems, Foster city, CA). Primers for hVEGF-A, Glyceraldehyde-3-phosphate dehydrogenase (GAPDH), and 18S rRNA (rRNA) were purchased from SABiosciences and had been verified by the provider. Human GAPDH and Human 18S rRNA were used as internal standards. The results were expressed as the n-fold expression of hVEGF, normalized on that of *GAPDH* and 18S RNA.

### Thiazolyl blue tetrazolium bromide assay

Ten thousand ARPE-19 cells per well were seeded in a 96-well plate and incubated overnight to allow the cells to attach to the plate. Thiazolyl blue tetrazolium bromide (MTT) was added into the media at the final concentration of 0.5 mg/ml for 4 h to allow MTT to be metabolized. Media were dumped off, and cells were resuspended in formazan (MTT metabolic product) in 200 µl dimethyl sulfoxide (DMSO). Optical density was read at 540 nm and background was substracted at 670 nm.

### Sodium dodecyl sulfate PAGE (SDS–PAGE) and western blotting

A total of 1 million ARPE-19 cells were lysed in 100 μl lysis buffer (50 mM Tris-Cl, 1% Triton X-100, 100 mM NaCl, 2 mM EDTA, 50 mM NaF, 50 mM glycerol-phosphate, 1 mM NaVO_4_, and 1× protease inhibitor cocktail [Roche Molecular Biochemicals, Indianapolis, IN]). Samples were prepared by adding an equal amount of 2× SDS protein loading buffer. Then the cells were vortexed and boiled at 95 °C for 5 min to achieve complete cell lysis. Cell debris was removed by centrifugation. Immunoblotting was performed according to the standard protocols. Primary antibodies of anti-phospho-protein kinase B (Akt), anti-phospho-Stat1, anti-Stat1, anti-phospho-p70 S6 kinase, phosphor-S6 ribosomal protein, and phospho-Eukaryotic translation initiation factor 4E-binding protein 1 (4E-BP1) were purchased from Cell Signaling Technology (Beverly, MA). Anti-β-actin antibody was from Santa Cruz Biotechnology, Inc. (Santa Cruz, CA). Anti–GAPDH antibody was from Abcam (Cambridge, MA).

### SiRNA transfection

Stat1 SiRNA (ON-TARGETplus SMARTpool) was purchased from Thermo Scientific (Lafayette, CO). *Akt* SiRNAs were from Cell Signaling Technology). The delivery of SiRNA to ARPE-19 cells was achieved by using DharmaFECT transfection reagents following the Thermo Scientific DharmaFECT General Transfection Protocol. After 48 h of transfection, the cells were stimulated with or without IFNγ.

### Measurement of hVEGF secretion from cells

Cell supernatants were analyzed for VEGF with the use of commercially available enzyme-linked immunosorbent assay (ELISA) kits (R&D Systems, Minneapolis, MN). The measurements were conducted according to the manufacturer's instructions. All samples were assayed in triplicates and the mean of the values was calculated.

### Statistical analysis

Analysis of hVEGF expression in cell supernatants was performed using the independent Student's *t*-test. In the figures, a single asterisk and a double asterisk indicate statistically significant at p<0.05 and p<0.01, respectively.

## Results

### IFNγ promoted VEGF expression of RPE cells

**Figure 1:**
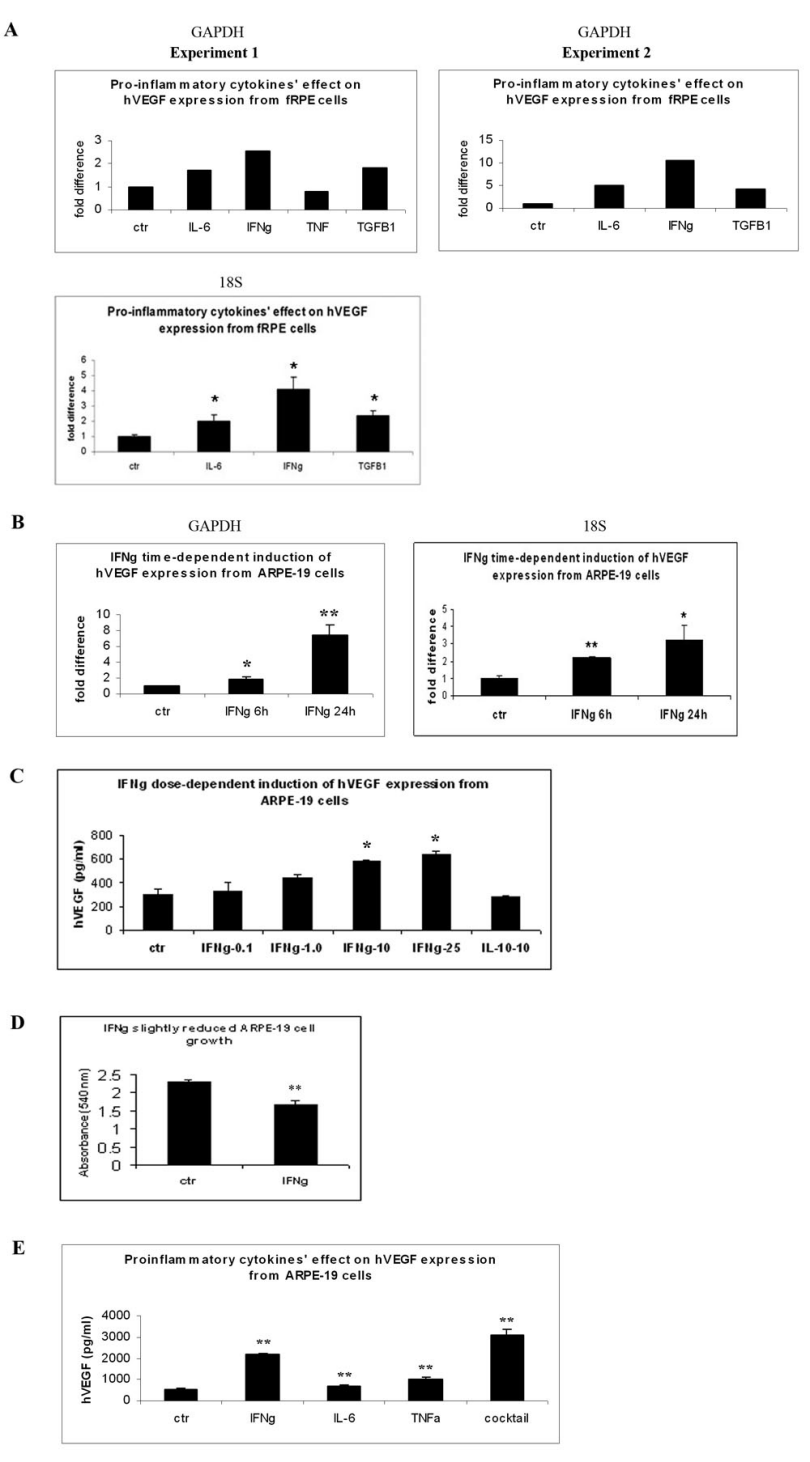
IFNγ-promoted vascular endothelial growth factor (VEGF) expression of RPE cells. **A**: fRPE cells were seeded in a 96-well plate and treated with IFNγ, IL-6, TNFα, and TGFβ1 overnight. In each experiment, each treatment was performed in triplicate. After 24 h of culture, cells were collected and pooled for RNA purification. Real-time PCR assay was performed, and the results were expressed as the n-fold expression of hVEGF normalized on that of GAPDH or 18S rRNA. The asterisk indicates statistical significance (p<0.05) compared to the non-treated control group. **B**: ARPE-19 cells were cultured with IFNγ for 6 h and 24 h. Cells were collected, and the RNA was purified for real-time PCR assay. The results were expressed as the n-fold expression of hVEGF normalized on that of GAPDH or 18S rRNA. The values are expressed as the average+SEM of triplicates for each treatment. The results were representative data from two separate experiments. The asterisk and the double asterisk indicate statistical significance (p<0.05 and p<0.01, respectively) compared to the non-treated control group. **C**: ARPE-19 cells were cultured with 0, 0.1, 1.0, 10.0, and 25 ng/ml IFNγ, as well as 10 ng/ml IL-10, for 48 h. Cell supernatants were collected and used for ELISA analysis. The y-axis represents hVEGF concentration (pg/ml). **D**: ARPE-19 cells were cultured with or without 25 ng/ml IFNγ for 48 h. MTT assay was used to detect cell viability. **E**: ARPE-19 cells were treated with IL-6, IFNγ, TNFα, and a cocktail of these three cytokines for 48 h. Cell supernatants were collected and used for ELISA analysis. The y-axis represents hVEGF concentration (pg/ml). The double asterisk indicates statistical significance (p<0.01) compared to the control group.

Because VEGF is one of the central regulators of vessel development, we were interested in testing whether pro-inflammatory cytokines could affect VEGF production and secretion from retinal cells. We used both primary fRPE and an adult RPE cell line, ARPE-19, to detect the effect of IFNγ on VEGF mRNA expression. We first treated fRPE cells with IL-6, IFNγ, TNFα, and TGFβ1 overnight. Cells were collected and RNA was purified for real time PCR assay using GAPDH as an internal standard. Compared to the control cells, IFNγ, IL-6, and TGFβ increased *VEGF* mRNA expression 2.56, 1.71, and 1.81 fold, respectively (Figure 1A, experiment 1). Another set of data showed a similar trend (Figure 1A, experiment 2). We also used 18S rRNA as an internal standard and found similar results. We then examined whether the same effect was also observed in ARPE-19 cells. As shown in Figure 1B, IFNγ started to induce *VEGF* expression at 6 h. At 24 h after treatment, IFNγ greatly induced *VEGF* RNA expression (p<0.01). Since we obtained similar results using both fRPE and ARPE-19 cells, considering that cell accessibility and adult RPE cells are more relevant to the pathological situation, we used ARPE-19 cells in the subsequent experimental settings. We next performed ELISA experiments to measure hVEGF secretion of ARPE-19 cells 48 h after cytokine treatment. As shown in the dose-dependent induction of hVEGF expression from ARPE-19 cells (Figure 1C), 1.0 ng/ml IFNγ started to induce hVEGF expression, with the highest effect at 25 ng/ml. However, the anti-proinflammatory cytokine, IL-10, did not affect hVEGF expression in ARPE-19 cells. MTT assay was used to detect cell number and viability after IFNγ treatment. As shown in Figure 1D, IFNγ slightly reduced ARPE-19 cell number and viability. We then compared the pro-inflammatory cytokines’ effect on VEGF expression using 25 ng/ml IFNγ, 10 ng/ml TNFα, and 50 ng/ml IL-6. ARPE-19 cells were treated with the above cytokines and a cocktail of these three cytokines. Cell supernatants were collected after 48 h and used for ELISA. As shown in Figure 1E, IFNγ, IL-6, TNFα, and the cytokine cocktail increased VEGF secretion 3.9, 1.22, 1.82, and 5.6 fold, respectively, higher than the controls (p<0.01). IFNγ had a more potent effect on VEGF secretion than did IL-6 or TNFα. The cytokine cocktail appeared to synergistically stimulate VEGF production from RPE cells.

### Stat1 activation is not involved in IFNγ-induced VEGF secretion

**Figure 2:**
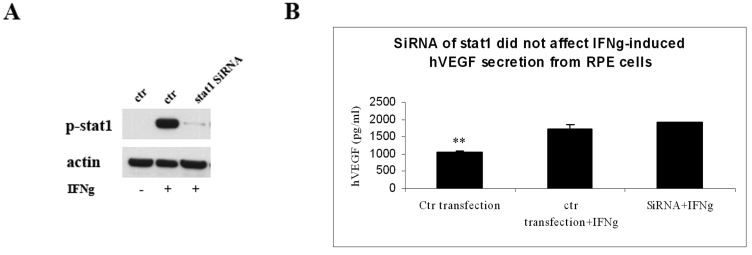
Stat1 activation not involved in IFNγ-induced VEGF secretion. **A**: ARPE-19 cells were transfected with SiRNA oligos targeting Stat1. After 3 days, cells were cultured with or without IFNγ for 30 min. Cells were then collected and processed for western blot analysis for p-Stat1 expression. The same blot was also stained with anti-β-actin antibody for loading control purposes. **B**: ARPE-19 cells were mock-transfected or transfected with SiRNA of Stat1. Two days later, cells were treated with or without IFNγ for 48 h. Cell supernatants were collected and used for ELISA analysis. The y-axis represents VEGF concentration (pg/ml). The values are expressed as the average+SEM of triplicates for each treatment. The double asterisk indicates statistical significance (p<0.01) compared to the IFNγ group.

Jak/Stat1 is the classic pathway IFNγ going through in signal transduction. We wanted to determine if this pathway is involved in IFNγ-induced VEGF secretion. We directly decreased Stat1 phosphorylation by transfecting SiRNA oligos targeting Stat1 into ARPE-19 cells. As shown in Figure 2A, compared to mock transfection, RPE cells with SiRNA of Stat1 had a dramatically lower expression of Stat1 phosphorylation. However, IFNγ-induced VEGF secretion showed no significant change between Stat1 SiRNA tansfected cells and control cells (Figure 2B).

### mTOR/translational pathway is involved in IFNγ-induced VEGF secretion from RPE cells

**Figure 3:**
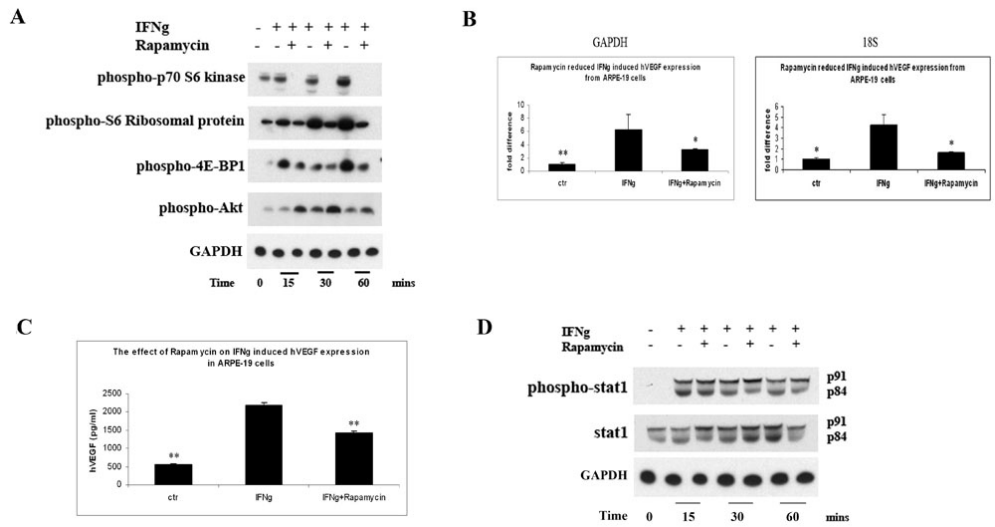
The mTOR/translational pathway was involved in IFNγ-induced VEGF secretion from RPE cells. **A**: ARPE-19 cells were cultured with or without IFNγ in the presence or absence of rapamycin for 15, 30, and 60 min. Cells were collected and processed for western blot analysis using anti-p-p70 S6 kinase, anti-p-S6 ribosomal protein, anti-p-4E-BP1, p-akt, and GAPDH antibodies. **B**: ARPE-19 cells were cultured with or without IFNγ/rapamycin for 24 h. Cells were collected for RNA purification. Real-time PCR assay was performed and the results were expressed as the n-fold expression of hVEGF normalized on that of GAPDH or 18S rRNA. **C**: ARPE-19 cells were cultured with or without IFNγ/rapamycin for 48 h. Cell supernatants were collected and used for ELISA analysis. The values are expressed as the average+SEM of triplicates of each treatment. The results were representative data from three separate experiments. The double asterisk indicates statistical significance (p<0.01) compared to the IFNγ group. **D**: ARPE-19 cells were cultured with or without IFNγ in the presence or absence of rapamycin for 15, 30, and 60 min. Cells were collected and processed for western blot analysis using anti-p-Stat1, Stat1, and GAPDH antibodies. The results were representative data from two separate experiments.

We assessed whether the mammalian target of rapamycin (mTOR) pathway was activated by IFNγ by determining the phosphorylation state of a series of downstream protein translation targets, such as p70-S6K1, S6 ribosomal protein, and 4E-BP1. As shown in Figure 3A, IFNγ dramatically increased the phosphorylation of these proteins after 15, 30, and 60 min of stimulation (see lanes 2, 4, and 6). Rapamycin greatly blocked the activation of the mTOR pathway by IFNγ (see lanes 3, 5, and 7). The role of mTOR in IFNγ-induced VEGF expression was evaluated. The administration of 10 nM rapamycin in the cell culture, at the same time as adding IFNγ, decreased VEGF mRNA expression (p<0.05, Figure 3B). Consistent with real-time PCR data, the ELISA results also demonstrated that IFNγ reduced VEGF secretion (p<0.01; comparing 2,181.5 pg/ml to 1,428.1 pg; Figure 3C). Rapamycin’s effect on the classic Jak-Stat1 pathway after activation by IFNγ was also tested. As shown in Figure 3D, rapamycin did not change IFNγ-induced Stat1 phosphorylation.

### PI3K/mTOR translational pathway is involved in IFNγ-induced VEGF expression

**Figure 4:**
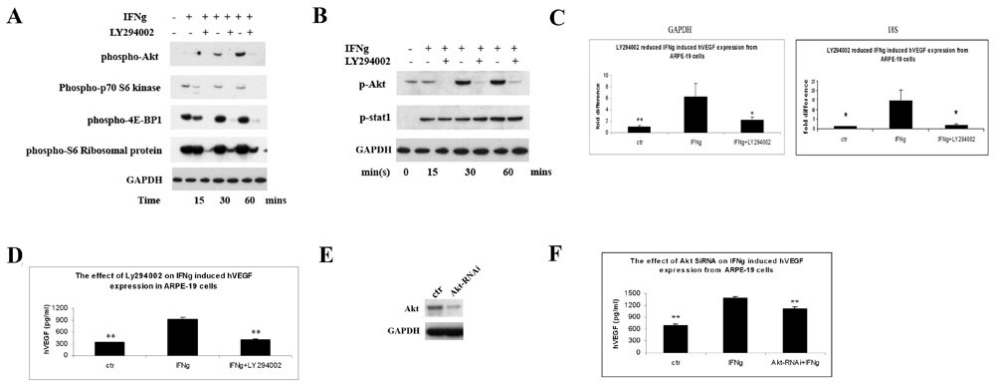
The angiogenic effect of IFNγ was through the PI-3K/mTOR translational pathway. **A**: ARPE-19 cells were cultured with or without IFNγ in the presence or absence of LY294002 for 15, 30, and 60 min. Cells were collected and processed for western blot analysis using anti-p-p70 S6 kinase, anti-p-S6 ribosomal protein, anti-p-4E-BP1, p-akt, and GAPDH antibodies. **B**: ARPE-19 cells were cultured with or without IFNγ in the presence or absence of LY294002 for 15, 30, and 60 min. Cells were collected and processed for western blot analysis using anti-p-akt, anti-p-Stat1, and GAPDH antibodies. The results were representative data from two separate experiments. **C**: ARPE-19 cells were cultured with or without IFNγ/LY294002 for 24 h. Cells were collected for RNA purification. Real-time PCR assay was performed, and the results were expressed as the n-fold expression of hVEGF normalized on that of GAPDH or 18S rRNA. **D**: ARPE-19 cells were cultured with or without IFNγ/PI-3K inhibitor LY294002 for 48 h. Cell supernatants were collected and used for ELISA analysis. The y-axis represents VEGF concentration (pg/ml). The values are expressed as the average±SEM of triplicates of each treatment. The double asterisk indicates statistical significance (p<0.01) compared to the IFNγ group. **E**: ARPE-19 cells were mock-transfected or transfected with SiRNA of akt. After 2 days, cells were then collected and processed for western blot analysis for akt expression. The same blot was also stained with GAPDH antibody as a loading control. The results were representative data from two separate experiments. **F**: ARPE-19 cells were mock-transfected or transfected with SiRNA of akt. Two days later, cells were treated with or without IFNγ for 48 h. Cell supernatants were collected and used for ELISA analysis. The y-axis represents VEGF concentration (pg/ml). The values are expressed as the average±SEM of triplicates of each treatment. The double asterisk indicates statistical significance (p<0.01) compared to the IFNγ group.

Multiple signaling pathways are activated during engagement of IFNγ with its receptor. Here, we used the PI3K inhibitor, LY294002, to detect if PI3K inhibition could affect IFNγ-induced activation of mTOR and its downstream molecules. Western blot staining showed that LY294002 completely blocked IFNγ-induced akt phosphorylation, confirming the inhibitory effect of PI-3K (Figure 4A). Furthermore, LY294002 diminished IFNγ-dependent downstream activation of the mTOR and p70 S6K pathways and the phosphorylation of 4E-BP1 (Figure 4A). However, LY294002 did not inhibit IFNγ-induced Stat1 tyrosine phosphorylation (Figure 4B). We then tested whether PI-3K activation was involved in IFNγ-induced hVEGF expression. As shown in Figure 4C, LY294002 greatly reduced hVEGF mRNA expression. The ELISA data (Figure 4D) also showed that LY294002 almost completely abrogated IFNγ-induced VEGF secretion from RPE cells (p<0.01; compare 923.2 pg/ml to 405.75 pg/ml. The control without adding IFNγ was 335.1 pg/ml.) To further address the role akt plays in IFNγ-induced VEGF expression, we knocked down the akt expression by transfecting SiRNA, targeting akt into ARPE-19 cells. As shown in Figure 4E, akt SiRNA dramatically decreased akt expression compared to mock transfection. Consistent with Figure 4C,D, akt SiRNA transfection greatly reduced the expression of hVEGF from ARPE-19 cells after IFNγ treatment (Figure 4F).

## Discussion

In the present study, we demonstrate that IFNγ induces VEGF secretion in RPE cells, and that this effect is dependent on the PI-3K/mTOR pathway. RPE is a single layer of epithelial cells in the back of the eye, acting as the outer blood-retinal barrier. Dysregulation of RPE has been implicated in AMD pathogenesis and ocular inflammation [17–20]. IFNγ is a crucial modulator of the immune system. It is produced by specific subsets of T lymphocytes and NK cells, and plays an important role in ocular pathogenesis [21,22]. IFNγ has a pleiotropic effect in the ocular environment. Not only is it involved in local inflammatory response, but it also magnifies this response in the outer retina. It upregulates the expression of MHC class II, ICAM-1, IL-6, IL-8, and MCP-1 in RPE cells, which collectively lead to alteration of RPE antigenic properties, and activation and recruitment of leukocytes in the retina, further contributing to ocular immunopathological processes [23–26]. Moreover, IFNγ is also reported to increase the expression of reactive oxygen species, which has been implicated in ocular inflammation and retinal degeneration [27]. The data from IFNγ transgenic animal models also support this notion. Geiger et al. found IFNγ transgenic mice developed intraocular disease, manifested as inflammatory-cell infiltration and photoreceptor loss [28]. Egwuagu and his associates also reported IFNγ transgenic mice had higher MHC class II expression in the eye, and the mice developed microphthalmia and microphakia [29]. The ectopic expression of IFNγ in photoreceptors disturbed the intraocular immune privilege in transgenic mice and prevented the induction of anterior chamber-associated immune deviation [30]. In IFNγ-transgenic rats, it has been reported that IFNγ accelerated the onset of experimental autoimmune uveitis (EAU) [31]. Interestingly, IFNγ also plays a protective role in regulating ocular immune response. Kim et al. [15] reported that IFNγ upregulated human CFH expression. CFH has been implicated in AMD pathogenesis and is an important component of downregulatory intraocular environment (DIE) [32]. Li et al. [33] found that application of IFNγ to the anterior surface of the rat eye could remove extra fluid in the subretinal region. This protective role in regulating retinal hydration may have implications for ocular pathogenesis, and more specifically, for macular edema-associated AMD and uveitis. In the mouse EAU models, it has been shown that IFNγ has a protective effect on EAU development, which contradicts the effects observed from human and rat EAU models [34]. Overall, the mechanisms of IFNγ function in different species need to be further explored.

Earlier reports emphasized that IFNγ is an anti-angiogenic cytokine. It inhibits the growth of endothelial cells (ECs) and induces the apoptosis of ECs [6]. In addition, treatment of ECs with IFNγ results in a decreased expression of the platelet EC-adhesion molecule (PECAM)-1, which is constitutively expressed by the vascular endothelium and concentrates at intercellular junctions [8,35]. The regulation of PECAM-1 expression by IFNγ is thought to affect leukocyte trafficking, angiogenesis, and vascular permeability. Moreover, IFNγ acts on ECs by inducing IFNγ-inducible protein 10 (IP-10) expression, which inhibits angiogenesis [36]. The proposed inhibitory effect of IFNγ on angiogenesis is not solely due to its direct activity on ECs. IFNγ differentially regulates IP-10 and IL-8 expression in RPE cells. Along with IL-1β and TNFα, IFNγ induced at least ten-fold higher IP-10 than IL-8, suggesting its direct anti-angiogenic effect [37]. Hooks and associates showed that IFNγ acts as an anti-angiogenic cytokine in the human cornea by inhibiting VEGFA and enhancing soluble VEGF-R1 secretion in human corneal stromal fibroblast cells [7]. Tumor cells respond to IFNγ by suppressing the expression of MMP-9 and MMP-2 genes, which are involved in the invasion and angiogenesis process of malignant tumors [38,39]. Betty et al. showed the inhibition of tumor angiogenesis by tumor-infiltrating CD4+ T cells to be IFNγ dependent [40]. Ray et al. reported that IFNγ suppresses VEGFA expression in monocyte-macrophages through translational silencing, by inducing the IFNγ-activated inhibitor of translation [41]. Furthermore, IFNγ has been shown to downregulate the expression of granulocyte-macrophage colony stimulating factor [42]. That and macrophage colony stimulating factor have been reported to be ideal targets for treating neovascular diseases and cancer [43,44].

In addition to its anti-angiogenic effect, there are also observations supporting its pro-angiogenic effects. An example is IFNγ’s reported angiogenic effects from enhancing the secretion of VEGF from human keratinocytes [45]. Shi et al. treated fRPE cells with IFNγ, IL-1β, and a TNFα cytokine cocktail. They found that a significant amount of cytokines and chemokines were upregulated, and that angiogenic cytokines were secreted at a greater rate than angiostatic cytokines [46]. Here, we are more focused on IFNγ’s effect on human RPE, because of the RPE’s central role in macular pathology involving the posterior pole. We therefore used human RPE cells as a model. For the first time, we have shown that although IFNγ slightly decreased ARPE-19 cell growth (Figure 1D), IFNγ induced VEGFA expression in ARPE-19 cells (Figure 1B,C). Compared to IL-6, TNFα, and TGFβ, IFNγ had a more robust VEGF-promoting effect at both the RNA and protein levels (Figure 1A,E), indicating that IFNγ is a versatile mediator in angiogenesis dysregulation associated with macular ocular pathology involving the back of the eye.

The anti-proliferative, proapoptotic effects of IFNγ have been largely attributed to the activation of the Jak/Stat1 signaling pathway [47–49]. However, several reports indicate that IFNγ activation of the Jak/Stat pathway is not sufficient to account for all the biologic actions of IFNγ [50–52]. Beyond the Jak/Stat1 pathway, IFNγ can also activate the MAPK and PI-3K/mTOR pathways [9]. However, the role of these pathways in IFNγ-induced biologic effects has not been clearly defined. In this study, we provide evidence that the PI-3K/Akt/mTOR/p70 S6 kinase pathway is important for IFNγ-induced VEGF expression in human RPE cells. The PI3K and mTOR inhibitors, along with Akt SiRNA, greatly decreased IFNγ-induced hVEGF secretion from RPE cells (Figure 3B,C and Figure 4C,D,F). On the other hand, neither inhibitor affected IFNγ-induced Stat1 phophorylation/activation (Figure 3D and Figure 4B). Moreover, the IFNγ-induced hVEGF expression was not affected by SiRNA targeted to Stat1 (Figure 2B), implying that the classic Jak/Stat1 pathway of IFNγ is not involved in this VEGF-promoting effect. The precise mechanisms that determine how the PI-3K/Akt/mTOR/p70 S6 kinase pathway affects VEGF expression remain unclear and need to be further explored.

We believe these observations have direct clinical implications. Even though the exact mechanisms leading to AMD remain obscure, early reports in the 1980s already suggested immune system involvement [53,54]. Recently, several genetic associations in humans [55] further implicate immune-driven mechanisms in AMD. mTOR is a crucial molecule in mediating inflammation-induced angiogenesis. In addition to IFNγ, which has been shown here to affect the pathway, other proangiogenic factors, including TNFα, IL-1β, IL-6, IL-8, basic fibroblast factor (bFGF), and VEGF, are dependent on the mTOR/translational pathway to contribute to the overall angiogenic process [56]. In line with this notion, an mTOR inhibitor, rapamycin, appears to be a suitable option to treat CNV that is secondary to any pathologic process involving the RPE, including AMD. In addition, rapamycin is also an immunosuppressant. It inhibits T-lymphocyte activation and proliferation, in response to both antigenic and cytokine stimulation [10]. The dual effects of this agent may be useful in treating the inflammatorily driven CNV. We are currently evaluating the use of rapamycin in treating AMD patients with CNV. The data suggest that rapamycin appears to affect the clinical course of AMD [data not shown]. The availabilities of IFNγ transgenic or knockout animal models may also help to elucidate the effect of rapamycin on ocular neovascularization. While we have emphasized the important role the mTOR/translational pathway plays in IFNγ-induced VEGF expression, we cannot exclude the possibility of other signaling pathways that are also important for this process. Kaur et al. [50] recently showed that PI-3K plays a dual regulatory role in IFNγ signaling by controlling IFNγ-dependent transcriptional regulation of IFN-sensitive genes, and by simultaneously regulating the subsequent initiation of mRNA translation for such genes. Consistent with their results, we found that IFNγ could promote VEGF mRNA expression, as well as protein secretion (Figure 1A,D).

Our findings provide further mechanistic insight into the pathways leading to CNV in disorders in which, like AMD, perturbation of RPE plays an integral role in disease pathogenesis. Our investigation’s elucidation of molecular signaling in IFNγ-induced VEGF expression provides further justification for therapeutic interventions to be carefully applied in the clinical setting.
